# Return to Play after SARS-CoV-2 Infection: Focus on the Pediatric Population with Potential Heart Involvement

**DOI:** 10.3390/jcm12216823

**Published:** 2023-10-29

**Authors:** Letizia Paglialonga, Camilla Aurelio, Nicola Principi, Susanna Esposito

**Affiliations:** 1 Pediatric Clinic, Department of Medicine and Surgery, University of Parma, 43126 Parma, Italy; letizia.paglialonga@gmail.com (L.P.); camilla.aurelio@unipr.it (C.A.); 2 Università degli Studi di Milano, 20122 Milan, Italy; nicola.principi@unimi.it

**Keywords:** cardiac involvement, COVID-19, heart, return to play, sport activities

## Abstract

The COVID-19 pandemic has entailed consequences on any type of physical activities, mainly due to the social restriction measures applied to reduce the spreading of SARS-CoV-2. When public health policies progressively reduced limitations and resuming a normal life was possible, the return to previous physical activity and sports was not only requested by people who had deeply suffered from limitations, but was also recommended by experts as a means of reducing the physical and psychological consequences induced by the pandemic. The aim of this narrative review is to summarize the available evidence on the return to play in children after SARS-CoV-2 infection, suggesting an algorithm for clinical practice and highlighting priorities for future studies. Criteria to identify subjects requiring laboratory and radiological tests before returning to physical activity are severity of COVID-19 and existence of underlying disease. Children of any age with asymptomatic infection or mild disease severity, i.e., the great majority of children with previous COVID-19, do not need a cardiologic test before resumption of previous physical activity. Only a visit or a telephonic contact with the primary care pediatricians should be established. On the contrary, children with moderate COVID-19 should not exercise until they are cleared by a physician and evaluated for resting electrocardiogram, exercise testing, and echocardiogram. Finally, in those with severe COVID-19, return to play should be delayed for several months, should be gradual and should be performed only after a cardiologist’s clearance. Further studies are needed to assess the risks of returning to sports activity in pediatric age, including careful age-adjusted risk stratification, in order to improve the cost–benefit ratio of specific screenings.

## 1. Introduction

Regular exercise and physical activity are essential for maintaining health. Sedentary behaviors have significant adverse impacts on the human body mainly because they are associated with an increased risk of development of cardiovascular diseases, metabolic disorders, cancer, musculoskeletal diseases, mental disorders and psychosocial misconducts [1,2]. Moreover, in children, sedentary behaviors are also associated with delayed and reduced physical growth and cognitive impairment [3]. The COVID-19 pandemic has entailed consequences on any type of physical activities, mainly due to the social restriction measures applied to reduce the spreading of SARS-CoV-2. People were obliged to remain at home, schools were closed, and sporting events were suspended. The overall physical activity levels dropped significantly worldwide, regardless of if it was light, moderate and/or vigorous before pandemic [4]. Several problems, particularly in adolescents and young adults, emerged. Together with several mental symptoms as well as anxiety, stress, depression, event-specific distress, decrease in psychological wellbeing, and changes in sleep habits [5], the limitation of physical activity and practicing sports led to an increased incidence of overweight, obesity and metabolic syndromes associated with a greater risk of cardiovascular diseases [6,7,8,9,10]. When public health policies progressively reduced limitations and resuming a normal life was possible, the return to previous physical activity and sports was not only requested by people who had deeply suffered from limitations, but was also recommended by experts as a means of reducing the physical and psychological consequences induced by the pandemic [11,12,13]. The aim of this narrative review is to summarize the available evidence on return to play in children after SARS-CoV-2 infection and potential heart damage, suggesting an algorithm for clinical practice and highlighting priorities for future studies.

## 2. COVID-19 and Sport Activities

As in the general population with COVID-19 cardiovascular problems had been frequently reported, physicians had to establish whether and how people that had suffered from this disease, including school-age children, adolescents, and collegiate and professional athletes, could return to previous life without any risk due to previous heart damage [14,15,16]. Initially, attention was mainly paid to elite athletes who placed high demand on their cardiorespiratory system. Careful health recommendations with a very conservative approach were prepared. Testing, including an ECG, measurement of cardiac troponin, and an echocardiogram for all athletes after COVID-19 infection was recommended [17,18,19]. Later, when more information regarding heart damage incidence and severity in COVID-19 patients was collected, less restrictive recommendations were prepared. They included, together with athletes, adults, adolescents, and children practicing non-professional sporting and physical activity of different intensity [20]. A problem raised by some experts was whether specific rules should be prepared for pediatric subjects who, in general, practice less intense physical activity than adults, especially professional athletes [21,22]. Pediatric guidelines were prepared detailing which children should require physician’s clearance before returning to play [23,24]. However, the comparison of adult and pediatric guidelines indicates that the same guidelines can be applied to both children and adults. The main difference is represented by the number of patients for whom heart tests and a physician’s clearance are requested before return to play. This because the risk of cardiological damage from COVID-19 between children and adults varies significantly. Unlike adults, children very often have asymptomatic COVID-19. Furthermore, the risk of myocardial damage in symptomatic cases is extremely low. Heart damage in the form of myocarditis, pericarditis, heart failure, cardiogenic shock, cardiac arrhythmia, and pulmonary hypertension, alone or in association with respiratory signs and symptoms, have been only sporadically reported in children during the acute phase of SARS-CoV-2 infection with death due to heart failure an exceptional event [25,26,27,28]. Moreover, although heart damage can be detected in up to 50% of children with the so-called multisystem inflammatory syndrome in children (MIS-C), this condition is very rare because it occurs in 1 case of MIS-C per 3164 cases of SARS-CoV-2 infection [29,30]. The same is true for the frequency of cardiovascular damage associated with the administration of COVID-19 vaccines, mainly the messenger RNA vaccines. A recent systematic review has estimated that the overall incidence of myopericarditis following COVID-19 vaccination is very low and is limited to 18 cases per million vaccine doses [31]. On the contrary, although the incidence of myopericarditis after receiving a COVID-19 vaccine is higher in adolescents and young adults than in older people, the reported frequency of cardiovascular involvement in adults with COVID-19 is in all the studies significantly higher, with the greatest incidence rates in patients with severe COVID-19 and in those with old age and severe chronic underlying disease favoring heart failure [32,33]. In a study enrolling 416 COVID-19 hospitalized adult patients, it was found that 20% had a myocardial injury and this was associated with a greater mortality than in patients without heart involvement (20% vs. 5%) [34]. Elevation of heart damage markers such as cardiac troponin and natriuretic peptide has been evidenced in up to 36% of the cases with a strict relationship between the elevation of markers and risk of heart failure and negative outcomes. Electrocardiographic and echocardiographic alterations were found frequently, as well as cardiovascular magnetic resonance findings suggesting myocarditis [34].

## 3. Main Guidelines about a Safe Return to Play

Consensus recommendations are constantly evolving, according to the acquisition of new data on the prevalence and the severity of cardiovascular sequelae related to COVID-19. The existing guidelines suggest algorithms based on risk stratification, categorizing patients according to the clinical presentation of COVID-19. Although official guidelines do not mention patients who have developed myopericarditis following COVID-19 vaccination, it seems obvious that children with vaccine-related heart damage should follow the same recommendations for returning to physical activity starting from the degree of the evidenced cardiac damage.

### 3.1. American College of Cardiology (ACC)

The first consensus statement endorsed by the ACC Sports and Exercise Cardiology Section, formulated for the adult population, recommended as return to play screening for all symptomatic athletes, the so-called triad testing with a 12-lead ECG, cTn (preferably using a high-sensitivity assay), and an echocardiogram [17]. Afterwards, the same group has recommended cardiac testing only in athletes with moderate, severe, and/or worsening symptoms caused by SARS-CoV-2 infection [35]. The most recent ACC guidelines classify patients based on the presence of symptomatology, time since infection, and severity of infection [36]. According to ACC recommendations, athletes with a recent SARS-CoV-2 infection that has passed asymptomatically may resume athletic activity after at least 3 days of abstention from physical activity during home isolation. In this group of patients, no further instrumental investigations are indicated. In individuals with mild to moderate infection in the absence of cardiovascular symptoms, a gradual return to physical activity is permitted after the resolution of symptoms. In athletes with a previous infection that has resolved for at least 3 months, a return to physical training is possible without performing further investigations.

A different approach is, on the contrary, recommended in case of individuals who have recovered from severe-critical SARS-CoV-2 infection or who have manifested symptoms suggestive of myocarditis or myocardial involvement, such as chest pain, palpitations, and syncope [17]. Triad testing (ECG, cTn, and echocardiogram) is indicated in this group of patients. The same investigations are also performed in those with new-onset cardiopulmonary symptoms after the resumption of sports activity. In case of triad test abnormalities or if cardiovascular symptoms persist, athletes should perform a cardiac magnetic resonance (CMR). According to existing guidelines formulated about myocarditis, athletes who recover from this cardiac complication should abstain from exercise for 3–6 months. In patients with persistence of cardiopulmonary symptoms, maximal-effort exercise testing and/or an ambulatory rhythm monitor may be helpful in the evaluation of athletes. Before performing these tests, myocarditis should be excluded with CMR. In athletes with recurrent COVID-19 without cardiopulmonary symptoms repeating cardiac testing is not warranted.

It would be incorrect to strictly apply adult guidelines to the pediatric population in all cases. In particular, it should be considered that most pediatric COVID-19 infections develop asymptomatically or pauci-symptomatically. Moreover, depending on the age, the intensity of physical exercise varies widely, from a recreational activity to competitive sports. Nevertheless, although it represents a rare event, cardiac injury from SARS-CoV-2 infection has been reported also in the pediatric population, and myocarditis represents one of the causes of sudden death during exercise in young athletic populations.

Considering these assumptions, the ACC has proposed an algorithm for the pediatric patient, which considers three aspects: how recent the infection is, the severity of the disease, and the type of physical activity to be returned to [37]. Even in the absence of clinical or laboratory evidence of cardiac involvement, before returning to play patients should be asymptomatic for at least two weeks, to rule out further clinical manifestations of COVID-19 and reduce the risk of virus transmission to other players. Mild SARS-CoV-2 infection takes off in the pediatric population in much the same way as other respiratory viruses. For this reason, as in the case of mild disease from other viruses, the guidelines recommend a gradual return to sports activity after the resolution of clinical symptoms. In patients with moderate symptoms, although they did not present with cardiac symptoms or perform cardiac testing during the acute phase, the presence of myocardial damage cannot be ruled out. In these subjects, a diversified approach is suggested depending on the age and intensity of physical exertion they are going to perform. Above the age of 12 years, it is recommended to perform a baseline ECG before returning to physical activity to detect signs of myocarditis. Depending on the situation, it may also be reasonable to perform 12-lead ECG, cTn (preferably using a high-sensitivity assay), and an echocardiogram, as suggested in adults. For patients less than 12 years old, since the intensity of exertion during sports is likely not significantly higher than their daily activities, in the presence of reassuring clinical history and exams, cardiac testing is not required.

In case of severe disease, defined as need of hospitalization, abnormal cardiac testing during the acute infection, and/or MIS-C, further investigations are needed before returning to play. The clinical course of patients with MIS-C is similar to that of myocarditis, so the same approach is suggested [17]. These patients will likely have had cardiac testing during the acute phase, such as ECGs and echocardiograms. Depending on the results of these, the patient should be restricted for 3–6 months and resume activities when cardiac testing (ECG, echocardiogram, 24 h Holter monitor, exercise stress test, and possibly cardiac magnetic resonance imaging) have normalized.

### 3.2. European Association of Preventive Cardiology

Recently, the European Association of Preventive Cardiology has proposed a protocol for return to play after SARS-CoV-2 infection that takes into account the need for a safe return to physical activities and also avoiding creating unnecessary barriers to physical activity, considering the beneficial effects of regular exercise in children’s physical, mental and cognitive health [23]. With this purpose, it has been suggested to base the cardiac evaluation before RTP on the severity of the disease and the presence of cardiac symptoms. Return to play should be gradual, after at least seven days after COVID-19. In asymptomatic individuals and those with mild symptoms, before return to play it is recommended to perform a physical examination and accurate evaluation of medical history by the physician. If symptoms are protracted or more than mild, also resting ECG, exercise testing, and echocardiogram should be performed, to exclude pericarditis and myocarditis.

In junior athletes that had severe cardiac manifestations or in case of abnormal clinical findings at the basal evaluation, cardiac magnetic resonance may be performed. Junior athletes diagnosed with pericarditis or myocarditis may return to play, respectively, after 1–3 months and after 3–6 months if they are asymptomatic and do not present abnormal clinical findings [23].

### 3.3. American Academy of Pediatrics (AAP)

Recently, the AAP has updated its guidelines on return to play after a SARS-CoV-2 infection in children [24]. To all the subjects who test positive for a SARS-CoV-2 infection, before resuming physical activity, at least one follow-up conversation or visit with their physician is recommended and should be performed within 2 to 4 weeks of a positive SARS-CoV-2 test. For those with asymptomatic infection or mild symptoms of COVID-19, AAP guidelines recommend an encounter with the primary care, in-person or even through telemedicine, by telephone interview, video call, or other digital communication. In these patients, if no issues emerge for attention, once isolation is completed, they can return to physical activity once medical therapy is completed and symptoms have resolved for at least one day. In preparation for the return to physical activity, the athlete and their parents should be educated about cardiac signs and symptoms suggestive of myocarditis, such as chest pain, shortness of breath out of proportion for upper respiratory tract infection, new-onset palpitations, or syncope. If these symptoms occur, stopping physical activity and performing a pediatric cardiology examination is recommended.

For patients with moderate symptoms of COVID-19, evaluation by the general practitioner is currently recommended, as these patients may be at an increased risk of cardiovascular sequelae [24]. A medical evaluation is needed after the resolution of symptoms and the end of the isolation period. It is recommended to perform the American Heart Association’s 14-item screening assessment, paying attention to cardiac symptoms, such as chest pain, shortness of breath not consistent with an upper respiratory tract infection, new-onset palpitations, or syncope. In addition to the evaluation of symptoms, a complete physical examination and an electrocardiogram should be performed. If the cardiac examination is negative, a gradual return to physical activity is possible 10 days after a positive COVID-19 test and after at least 1 day of no symptoms (excluding loss of taste/smell) and discontinuation of antipyretic therapy. If abnormal elements emerge from this initial assessment, a pediatric cardiological examination is recommended. The cardiologist may deem it necessary to perform an echocardiogram, a serum troponin assay, and, in selected cases, more investigations, such as Holter ECG monitoring, a stress test, or a cardiovascular magnetic resonance (CRM). Also in this case, it is important to provide all athletes and their parents with indications for monitoring the signs and symptoms of myocarditis upon a return to physical activity. In the case of the onset of warning symptoms, it is recommended that physical activity be discontinued and that further investigations be carried out by a pediatric cardiologist.

For children and adolescents with a severe form of COVID-19 (ICU stay and/or intubation) or MIS-C, it is recommended to limit physical activity for a minimum of 3–6 months prior to resuming training or competition [24]. Authorization from the cardiologist is required before resuming physical activity. In the case of subjects with a history of SARS-CoV-2 infection who have already independently returned to physical activity, if no abnormal signs or symptoms are present, no further investigations are recommended.

### 3.4. Italian Sports Medical Federation (FMSI)

In agreement with the ACC guidelines, the FMSI has proposed an update of applicable guidelines and recommendations regarding eligibility for competitive sports activity in athletes infected with SARS-CoV-2 [38]. These guidelines divide subjects based on the clinical presentation from SARS-CoV-2 infection into three categories: patients who presented with asymptomatic/paucisymptomatic infection or mild disease, patients with moderate disease, and patients with a severe or critical presentation. In young athletes with a history of asymptomatic/paucisymptomatic infections or mild disease, medical evaluation is indicated, considering concomitant diseases at cardiovascular risk and anti-SARS-CoV-2 vaccination status. For this first category of patients, it is suggested, in addition to the medical examination carried out by the specialist in Sports Medicine, to perform the following diagnostic tests: ECG and exercise test with continuous electrocardiographic monitoring (also with step-test) until at least 85% of max HR is reached, which in patients with a positive history of pathologies identified as cardiovascular risk factors is replaced by a maximal incremental ergometric test with electrocardiographic monitoring. In the absence of cardiovascular risk pathologic conditions, in patients who have received a booster dose or completed the vaccine course or have recovered from SARS-CoV-2 infection within the previous 120 days, these tests should be performed once at least 7 days have elapsed since recovery from SARS-CoV-2 infection. In patients with cardiovascular risk diseases or who have not received dose boosters or recovered from SARS-CoV-2 infection in the previous 120 days, the above examinations should be performed no earlier than 14 days after recovery. In the case of professional athletes of national/international interest, the protocol provides for a quicker return to sports activity immediately after recovery by performing, in addition to the clinical evaluation carried out by the Sports Medicine specialist, the following instrumental investigations: baseline ECG, Maximal Incremental Ergometric Test with electrocardiographic monitoring, Color Doppler Echocardiogram.

In the other two groups of subjects (i.e., those who recovered from moderate SARS-CoV-2 and critical-severe disease, respectively), a return to play is possible at least 30 days after recovery [38]. In the two categories, a specialized clinical evaluation performed by a medical sport specialist and the following additional diagnostic investigations are indicated: maximal incremental ergometric test with electrocardiographic monitoring and measuring of O_2_ saturation at rest, during, and after the test; Color Doppler echocardiogram; 24 h Holter ECG including training or exercise session; spirometric examination; hematochemical examinations (i.e., complete blood count, ALT/AST, gamma GT, creatinine, CPK cardiac isotypes, LDH, PT/PTT, INR, protein electrophoresis, C reactive protein, ferritin, complete urine examination). In individuals with severe or critical illness, the cardiopulmonary exercise test (CPET) is recommended in addition to the tests described above. In these groups of patients, it is also within the discretion of the evaluating physician to request further investigations or additional specialist visits based on organ involvement.

The FMSI guideline is the only one that included a large number of exams for children recovered from moderate to severe COVID-19, leading to performing several diagnostic tests that would not be necessary. A study conducted by the Institute for Maternal and Child Health of Trieste, Italy, evaluated pediatric patients before their return to sports activity, subjecting them to screening proposed by the FMSI [39]. The study showed that none of the patients with a history of asymptomatic or mild symptomatic SARS-CoV-2 infection presented cardiac or pulmonary function limitations, and all patients enrolled in the study received eligibility for return to play.

## 4. Conclusions

In all the available guidelines, the criteria to identify subjects requiring laboratory and radiological tests before returning to physical activity were severity of COVID-19 and existence of underlying disease. Considering that children have a significant lower risk of severe COVID-19 than adults, rigid screening strategies to identify subjects at risk of heart problems and physician’s clearance should be considered mandatory for a significantly lower number of cases. This explains why, according to the American Academy of Pediatrics [24] and the European Association of Preventive Cardiology [23], children of any age with asymptomatic infection or mild disease severity, i.e., the great majority of children with previous COVID-19, do not need cardiologic tests before the resumption of previous physical activity. Only a visit or a telephonic contact with the primary care pediatricians should be established. Parents should be advised to monitor the child for the development of signs/symptoms suggesting heart problems and, in this case, to stop physical activity and consult a cardiologist. On the contrary, children with moderate COVID-19 should not exercise until they are cleared by a physician and evaluated for resting electrocardiogram, exercise testing, and echocardiogram. Finally, in those with severe COVID-19, a return to play should be delayed for several months, should be gradual and should be performed only after a cardiologist’s clearance.

**Figure 1:**
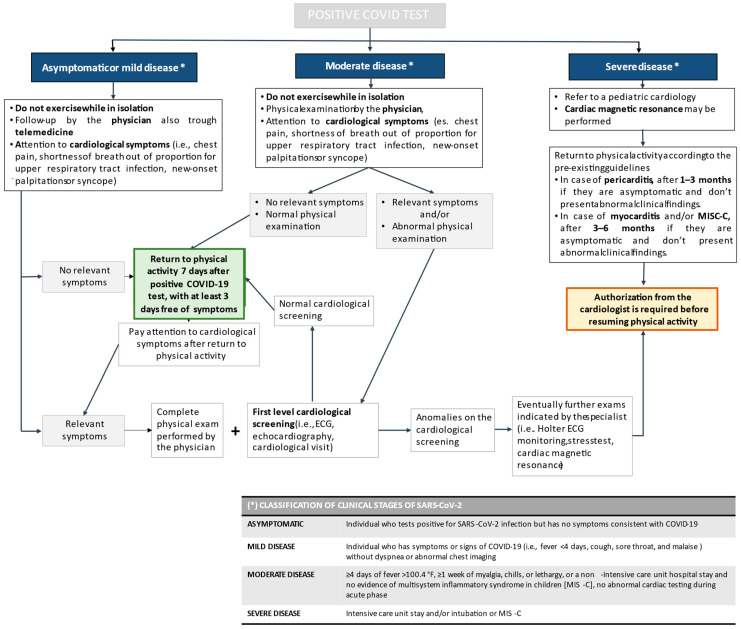
Recommended algorithm for return to play in children with previous SARS-CoV-2 infection.

Figure 1 shows our recommended algorithm for return to play in children with previous SARS-CoV-2 infection. However, further studies are needed to assess the risks of returning to sports activity in pediatric age, including careful age-adjusted risk stratification, in order to improve the cost–benefit ratio of specific screenings.

## Data Availability

Not applicable.
